# Paclobutrazol Residue Determination in Potato and Soil Using Low Temperature Partition Extraction and Ultrahigh Performance Liquid Chromatography Tandem Mass Spectrometry

**DOI:** 10.1155/2015/404925

**Published:** 2015-09-10

**Authors:** Hongcheng Liu, Tao Lin, Jia Mao, Huan Lu, Dongshun Yang, Jiliang Wang, Qiwan Li

**Affiliations:** ^1^Supervision & Testing Center for Farm Product Quality, Ministry of Agriculture, Kunming, China; ^2^Laboratory of Quality & Safety Risk Assessment for Agro-Product, Ministry of Agriculture, Kunming, China; ^3^Institute of Quality Standard and Testing Technology, Yunnan Academy of Agriculture Science, Kunming 650223, China; ^4^Institute of Agriculture Environment and Source, Yunnan Academy of Agriculture Science, Kunming 650223, China; ^5^Kunming Medical University, Kunming, China

## Abstract

A simple, accurate, and highly sensitive analytical method was developed for determining the paclobutrazol residue in potato and soil, the dynamics dissipation in soil. Extraction was carried out by low temperature partitioning and analyzed by ultrahigh performance liquid chromatography tandem mass spectrometry (UHPLC-MS/MS). For a favor extraction yield, the parameters such as temperature and solvent were optimized. The result showed that sample would be easily frozen and separated using acetonitrile under −20°C for 10 min. The limit of detection (LOD) was 0.5 *μ*g/kg, and the limit of quantification (LOQ) was 2 and 5 *μ*g/kg for potato and soil, respectively. The influence of paclobutrazol residue in potato was evaluated. The possible contamination of paclobutrazol from surface can be rinsed by distilled water or peeled off, but the paclobutrazol in potato harvest comes mainly from absorption and transport, which could not be removed by peeling. The half-life of paclobutrazol in soil was 20.64 days, and the residue was below 0.22 mg/kg on 50th day after spraying. According to the risk assessment with Need Maximum Daily Intake (NEDI) and Acceptable Daily Intake (ADI), a Maximum Residue Limit (MRL) of paclobutrazol in potato was recommended as 1.0 mg/kg.

## 1. Introduction

**Figure 1:**
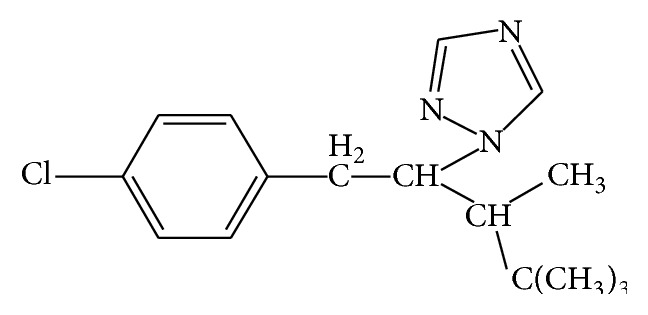
The chemical constructure of paclobutrazol.

Paclobutrazol (chemical structure as in Figure 1) was a plant growth regulator registered for the reduction of terminal growth and pruning volume, the inhibition of gibberellins and sterol biosynthesis, and hence the rate of cell division [1, 2]. Due to its toxicity, the agreed ADI (Accepted Daily Intake) and ARfD (Acute Reference Dose) were all 0.1 mg/kg bw/day. According to Reg. number 396/2005 (EC) Annex I, the MRL for paclobutrazol in fruit was 0.5 mg/kg, and the national food safety quality standard (GB 2763-2014, China) was 0.5 mg/kg in wheat and rice. However, no information was available concerning the residual status and pattern of paclobutrazol in potato. Growth inhibition characteristics had been reported for paclobutrazol with soil drenches, soil sprays, and foliar sprays. Cross-comparisons among these studies were difficult since they were conducted under different growth conditions (field, greenhouse, and growth chamber). Two other factors that influenced the paclobutrazol residues were absorption rate and longevity or persistence of the compound in treated plant tissue and soil [3].

Traditionally, gas chromatography (GC) and gas chromatography-mass spectrometry (GC/MS) was once popular for analyses of paclobutrazol [4], but GC/MS required prederivatization and target compounds may be decomposed by injection at high temperatures [5]. More recently, HPLC-based methods for analyses of paclobutrazol had been published in plant [6–9] and pear pulps [10] using UV detection and different cleanup steps in order to get recoveries of 70% at the 0.01 mg/kg level [10]. Thus, using these conventional techniques, it was necessary to perform one or more cleanup steps to decrease interferences and preconcentration steps in order to obtain adequate detection levels.

Nowadays, LC coupled to tandem MS (MS-MS) [11–13] had also been applied to plant growth regulator analysis in fruits as a powerful confirmation tool, improving the sensitivity and reducing the sample pretreatment steps, such as solid phase extraction (SPE) techniques.

Recently, the liquid-liquid extraction with low temperature partitioning (LLE-LTP) was promising for multiresidues analysis since this technique presented some advantages in relation to other extraction techniques, such as practicability, reduced number of steps, and low consumption of organic solvents, as well as being reliable and selective [11, 14].

In this study, it was to develop and validate a UHPLC-MS/MS method for determination of paclobutrazol in potato and soil, taking advantage of liquid-liquid extraction at low temperature. In addition, the paclobutrazol residue in potato and soil and the dynamics dissipation in soil were investigated through field trials. The purpose of this study was to evaluate the influence of paclobutrazol residue in potato, and a MRL of paclobutrazol in potato was recommended.

## 2. Experimental

### 2.1. Chemicals and Reagents

A High-Performance Liquid Chromatography- (HPLC-) grade acetonitrile and methanol were obtained from Merck Co. Ltd. (Germany). Sodium chloride and sodium acetate of analytical grade were obtained from the Chemical Reagent Company (Shanghai, China). Highly purified water (Milli-Q, Millipore, Bedford, MA) was used throughout the preparation of the mobile phase.

Paclobutrazol of certified standards (purity higher than 95%) was purchased from Dr. Ehrenstorfer GmbH (Augsburg, Germany). Stock solution was prepared at 1000 mg/L in methanol and stored in dark vials at −20°C. For the calibration curve, matrix-matched calibration was used with five series concentrations of extraction solution (0.001, 0.01, 0.05, 0.1, and 0.5 mg/L).

### 2.2. Experimental Design

To investigate the MRL of paclobutrazol in potato and soil, ultimate residue experiments were conducted in experimental fields located in Songming agriculture demonstration garden, Yunnan Province, China, from April 18 to June 29, 2013. The soil was sandy clay loam, with content of sand 46%, clay 28%, organic matter 2.02%, and pH 6.94. Fields were divided into 30 m^2^/plot with buffer areas. Each treatment was replicated for 3 plots. All cultural practices were applied according to the regional recommendation. The experiments were designed according to NY/T 788–2004 (Guideline on Pesticide Residue Trials) issued by the Ministry of Agriculture, China.

According to the usage guide, paclobutrazol formulation spray application was recommended one time during the early flowering stage of potato (in 45 days). Plants were treated with 3 dosage levels, 40 g (available ingredient)/ha (the low dosage, recommended dosage), 60  g (available ingredient)/ha (the middle dosage, 1.5 times the recommended dosage), and 100 g (available ingredient)/ha (the high dosage, 2.5 times the recommended dosage). Treatments were consisted of foliar sprayed (with or without thick polypropylene soil cover), and the tuber on the paclobutrazol residual soil with 60 g (available ingredient)/ha, and the control plants were treated with distilled water. Foliar sprays were applied with and without a ground cover to evaluate the impact of foliar adsorption and indirect soil absorption from foliar runoff on soil residue.

To check the possibility of paclobutrazol contamination from surface and peel of tuber, twenty tubers (4.8 kg) were sprayed with paclobutrazol (1.0 mg/L), five of which were rinsed with 500 mL distilled water and 100 mL acetonitrile; then the rinsing acetonitrile was determined by UHPLC-MS/MS after 30 min. Another five samples were peeled; then the peel and pulp were analyzed for comparison with potato harvest.

As reported paper [15], potato was harvested about one month after flowering stage, so the tuber and soil were collected to determine final residue at ten days before harvest (22 days), harvest time (32 days), and eight days after harvest (40 days) after foliar spraying.

To determine dissipation rate of paclobutrazol in soil, nine soil samples were collected after spraying. Soil samples of approximately 500 g from depth of 0–10 cm were collected randomly from five points in each plot at 1 h, 1, 3, 7, 14, 21, 32, 40, and 50 days after soil spray.

### 2.3. Sample Preparation

The potatoes peeled were smashed into pieces with a vegetation disintegrator after simple cutting. The soil samples were air-dried, clod broken, mixed, and sieved through a 2 mm sieve as described by Sharma [16]. These samples were stored at −20°C before being analyzed.

### 2.4. Extraction Procedure

Soil: 10 g soil sample was weighed into a 100 mL centrifuge tube and then 50 mL acetonitrile was added. The centrifuge tube was extracted in an ultrasonic bath for 10 min and centrifuged at 3000 g for 5 min. Afterwards, the tube was stored at low temperature (−20°C) in refrigerator (Haier, Qingdao, China) for 10 min to easily separate organic layers. Two-milliliter portions of the organic layer were filtered through 0.2 *μ*m membrane filters prior to analysis by UHPLC-MS/MS.

Potato: 25 g sample was weighed into a 100 mL centrifuge tube and extracted with 50 mL acetonitrile. The same procedure was followed as that described for soil up to UHPLC-MS/MS.

### 2.5. UHPLC-MS/MS Detection

Sample analyses were performed on tandem mass spectrometry AB 4000 (AB Sciex, Ontario, Canada) which consisted of a 1290 ultrahigh performance liquid chromatography (Agilent Technology, USA). A Zorbax Eclipse Plus C18 column (50 mm × 2.1 mm i.d., 1.8 *μ*m particle size) was employed for the separation of the analyte and was maintained at 30°C. The mobile phase was comprised of acetonitrile/water containing 0.1 percent formic acid (78/22,* v/v*) and was delivered at a constant flow of 0.3 mL/min. The injection volume was 1 *μ*L. The spectral acquisition was operated in positive electron spray ionization mode, and multiple reactions monitoring (MRM) was utilized. The gas temperature was set at 350°C with a flow rate of 8.0 L/min. The nebulizer pressure was 10 Pa. The precursor ion was* m/z* 294.2, and production ion was* m/z* 129.0, 70.0 for paclobutrazol, with relative intensity of 4.5%/68%, in which the most intense production ion* m/z* 70.0 was the quantitative ion, and collision energy and fragmentor were 110 and 50 eV, respectively.

## 3. Results and Discussion

### 3.1. Sample Extraction and Analytical Procedures

Appropriate sample preparation influenced the reliability of the obtained results in a significant way. Solid-Phase Extraction (SPE) was a preferred way to prevent matrix interference, but it was time-consuming with high cost [14]. The advantage of liquid-liquid extraction with low temperature was that the sample components remained in the ice phase, whereas paclobutrazol was extracted by the completely transparent liquid, which was easily obtained and directly analyzed by UHPLC-MS/MS.

As it had been previously indicated, Cho et al. [11] had studied in detail the low temperature extraction of pesticide under −80°C. The result showed that the sample would be frozen and separated easily under −20°C to 10 min. So it can be operated under facile condition by home refrigerator.

In order to gain a favorable extraction yield, various organic solvents were studied. The extraction yield of the various solvents for paclobutrazol was listed in the following sequence: acetonitrile (recovery 95%) > acetone (recovery 86%) > dichloromethane (recovery 65%) > *n*-hexane (recovery 10%). So the optimal solvent was acetonitrile. Furthermore, the ratio of acetonitrile to the sample showed that the best results were with 2 : 1 (potato) and 5 : 1 (soil), resulting in 85% recovery greater than other ratios with 1 : 1 or less.

The tuning solution was introduced into the electrospray ionization (ESI) source by direct infusion. The main ions produced in MS and MS/MS were identified in positive ionization modes. The obtained precursor ions indicated a clear relation between the structure of paclobutrazol and its ability for positive ionization. The precursor ion was* m/z* 294.2, and production ion was* m/z* 294.2–129.0 and 294.2–70.0 (quantitative ion).

**Figure 2:**
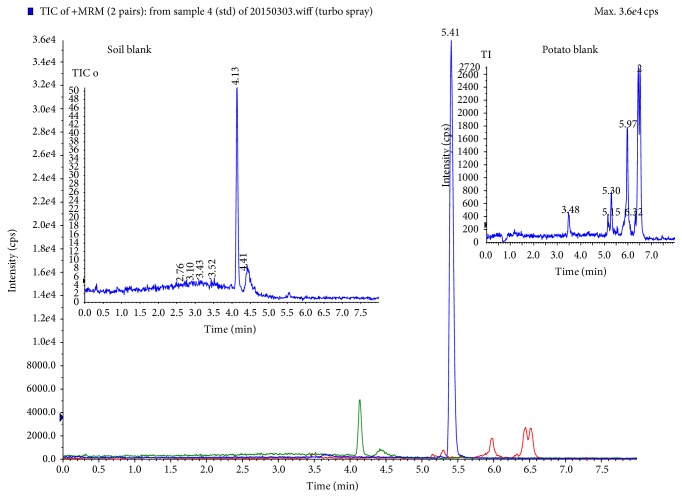
Multiple reaction monitor (MRM) chromatograms of paclobutrazol fortified and potato blank and soil blank in quantitative ion, *t* = 5.41 min (paclobutrazol).

The composition of mobile phase can influence the performance of the ionization process in ESI mode. So, different mobile phases (acid, base, and neutral) were tested. The result showed that the response of paclobutrazol can be increased in acid mobile phase (0.1% formic acid). The optimized mobile phase was acetonitrile/water containing 0.1% formic acid (78/22,* v/v*), as shown in Figure 2.

### 3.2. Valid Method

The matrix effects will suppress the ionization efficiency of paclobutrazol and low recovery. For this reason, residues of paclobutrazol in samples were quantified with matrix-matched five-series standard calibration (0.001 mg/L, −0.5 mg/L). The calibration curves showed good linearity with the following equations and relative coefficients: *y* = −1.95693 × 10^6^ + 964964*x*, correlation coefficient *R* ^2^ = 0.9976. The instrument detection limit (LOD) and limit of quantification (LOQ) were estimated through ten repetitive injections of standard solution, which can detect at a signal-to-noise ratio (*S/N*) of three multiples and ten multiples, respectively. The LOD value was 0.5 *μ*g/kg, and the LOQ values were always 2 *μ*g/kg in the potato and 5 *μ*g/kg in soil. The result of residue experiment can be guaranteed by the LOQ. Samples with concentrations lower than the LOQ were considered not quantifiable. Repeatability and reproducibility were expressed as relative standard deviations (RSD) of retention time (Rt) and peak area (Ar). Reproducibility of Rt and peak area was good for 1 month; all values were 3.4% (Rt) and 5.2% (peak area) or less. Furthermore, repeatability of Rt and peak area, which was evaluated on 1 day, proved more satisfactory, with values of 1.5% (Rt and peak area) or less.

The accuracy of the whole method was evaluated by the development of a recovery study carried out at three concentration levels (5, 50, and 100 *μ*g/kg). All experiments were carried out in quintuplicate at each level (results are shown in Table 1). As it can be seen in Table 1, recovery values were satisfactory, ranging between 83 and 106% with RSD lower than 10%. As it can be seen from the RSD values, the method was reproducible and applicable to the analysis of paclobutrazol in potato and soil.

### 3.3. Influence of Paclobutrazol Residue in Potato

The residue values of paclobutrazol in potato and soil were listed in Table 2. Foliar spray without cover treatment showed that amounts of paclobutrazol were absorbed by foliage and foliar runoff was absorbed by the soil. Mauk et al. [17] showed result that paclobutrazol residues in the soil increase due to falling leaves from nearby sprayed trees. Apparently, the amount of paclobutrazol absorbed by stolon, then transported to tuber, was larger than by foliar absorptive capacity.

**Figure 3:**
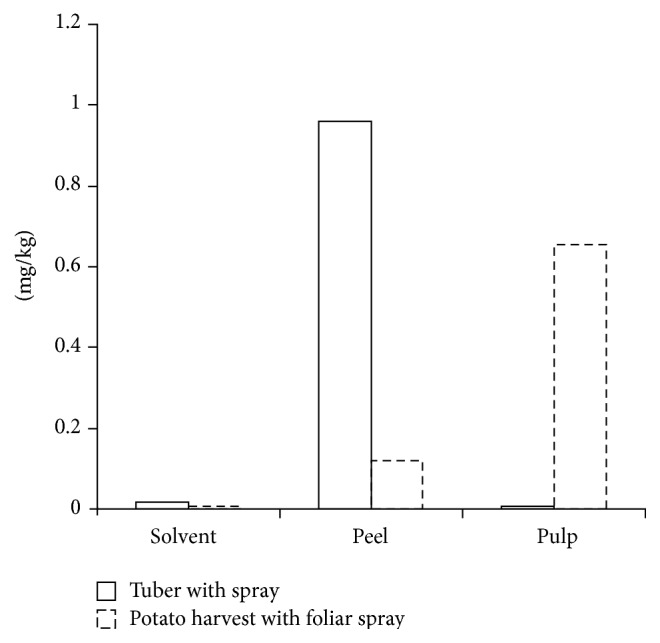
The content of paclobutrazol with different treatment after peeling and rinsing.

To check the possible contamination of paclobutrazol from surface and peel of tuber, the content of paclobutrazol in different treatment was shown in Figure 3. The content in organic solvent was very lower than the expected concentrations in whole potato. The paclobutrazol on surface can be rinsed by distilled water or peeled. So the paclobutrazol residue in potato harvest was mainly from absorption and transport from soil, which was not removed by peeling.

### 3.4. Dissipation Rate of Paclobutrazol in Soil

**Figure 4:**
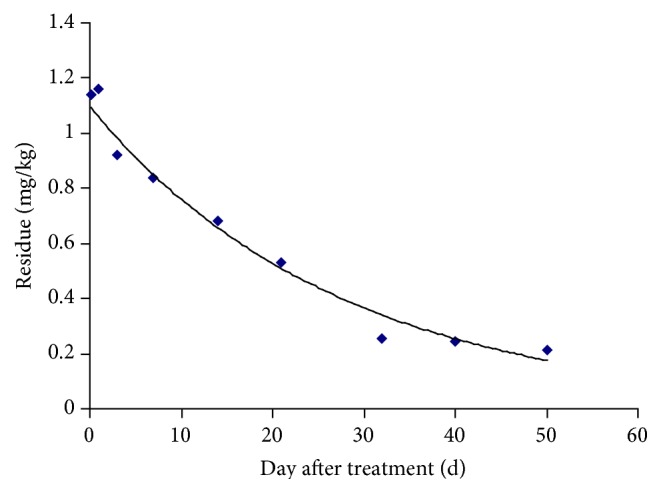
The dynamic dissipation rate of paclobutrazol in soil.

The dissipation curve of paclobutrazol in soil was shown in Figure 4. The result showed that the initial deposits of paclobutrazol in soil were 1.14 mg/kg. Except for a slight increase on 1st day, the residue value showed a steady decrease and was below 0.22 mg/kg on 50th day. The half-lives calculated were 20.64 days. The dissipation equation of paclobutrazol was *y* = 1.0784*e* ^−0.037 d^, *R* ^2^ = 0.9403.

### 3.5. Risk Assessment of Paclobutrazol in Potato

To establish a recommended Maximum Residue Limit (MRL) of paclobutrazol in potato, two guideline values, with Need Maximum Daily Intake (NEDI) and Acceptable Daily Intake (ADI), were needed. The value of ADI with paclobutrazol was 0.1 mg/kg bw/day. NEDA was derived on the supervised trials median residue (STMR) or processing factor (STMR-P) and food intake rate (*F*). Finally, risk assessment was performed to ensure that the established “safe” level of exposure (MRL) does not exceed the estimated level of actual exposure (ADI).

The equation was

(1) $$\begin{array}{c} \text{NEDI}=\sum \left[\;{\text{STMR}}_{i}\left(\;\text{STMR-}P_{i}\;\right)\times F_{i}\;\right]. \end{array}$$

According to the investigation of nutrition and health by Chinese Health Ministry in 2002, the *F* of potato was 0.0496 kg, STMR in potato harvest (recommended dosage: high dosage level) was 0–0.521 mg/kg, and then the high value NEDI (0.0258 mg) was not beyond 5% the value ADI × 63 (adult weight) of 6.3 mg. Therefore, a MRL in potato was recommended as 1.0 mg/kg.

## 4. Conclusion

Liquid-liquid extraction with low temperature partitioning was developed and validated for analysis of paclobutrazol in potato and soil using UHPLC-MS/MS. The optimal chromatographic separation and sensitivity were successfully applied to the analysis of paclobutrazol residue in potato and soil. According to risk assessment with NEDI and ADI, a MRL of paclobutrazol was recommended as 1.0 mg/kg.

## Figures and Tables

**Table 1:** Recoveries from two samples (five replicates).

Sample	Spiked value (*μ*g/kg)	Paclobutrazol	
		RSD (%)	Recovery (%)
Potato	5	7.89	106.3
	50	6.54	87.5
	100	3.62	93.0

Soil	5	10.1	104.9
	50	6.51	82.9
	100	8.76	83.4

**Table 2:** Final residues of paclobutrazol in potato and soil.

Sample	Treatment	Dosage g (available ingredient)/ha	Residues (mg/kg ± SD, *n* = 3), day after spraying		
			22 d	32 d	40 d
Potato	Foliar spray without cover	40	ND^a^	ND	ND
		60	0.041 ± 0.012	0.022 ± 0.008	0.017 ± 0.006
		100	0.732 ± 0.028	0.521 ± 0.022	0.481 ± 0.017
	Foliar spray with cover	40	ND	ND	ND
		60	ND	ND	ND
		100	ND	ND	ND

Soil	Foliar spray without cover	40	0.081 ± 0.019	0.032 ± 0.013	0.021 ± 0.006
		60	0.517 ± 0.027	0.258 ± 0.021	0.224 ± 0.017
		100	2.71 ± 0.23	1.42 ± 0.17	1.08 ± 0.12
	Foliar spray with cover	40	ND	ND	ND
		60	ND	ND	ND
		100	ND	ND	ND

^a^ND: not detected.
